# Preserving mitochondrial function by inhibiting GRP75 ameliorates neuron injury under ischemic stroke

**DOI:** 10.3892/mmr.2022.12681

**Published:** 2022-03-15

**Authors:** Bin Wen, Kai Xu, Rui Huang, Teng Jiang, Jian Wang, Jiehui Chen, Juan Chen, Benhong He

**Affiliations:** 1 Department of Biochemistry and Molecular Biology, School of Basic Medicine and the Collaborative Innovation Center for Brain Science, Tongji Medical College, Huazhong University of Science and Technology, Wuhan, Hubei 430030, P.R. China; 2 Department of Cardiovascular Medicine, Lichuan People's Hospital, Lichuan, Hubei 445400, P.R. China

**Keywords:** GRP75, calcium overload, mitochondria-associated endoplasmic reticulum membrane, mitochondria

## Abstract

Ischemic stroke is a life-threatening disease, which is closely related to neuron damage during ischemia. Mitochondrial dysfunction is essentially involved in the pathophysiological process of ischemic stroke. Mitochondrial calcium overload contributes to the development of mitochondrial dysfunction. However, the underlying mechanisms of mitochondrial calcium overload are far from being fully revealed. In the present study, middle cerebral artery obstruction (MCAO) was performed *in vivo* and oxygen and glucose deprivation (OGD) *in vitro*. The results indicated that both MCAO and OGD induced significant mitochondrial dysfunction *in vivo* and *in vitro*. The mitochondria became fragmented under hypoxia conditions, accompanied with upregulation of the heat shock protein 75 kDa glucose-regulated protein (GRP75). Inhibition of GRP75 was able to effectively ameliorate mitochondrial calcium overload and preserve mitochondrial function, which may provide evidence for further translational studies of ischemic diseases.

## Introduction

Ischemic stroke is a problematic health issue worldwide. The major pathophysiological interference of ischemic stroke is the sudden obstruction of cerebral arteries, which immediately causes neurons to suffer from hypoxia (1,2). As a highly energy-dependent organ, the brain is vulnerable to hypoxia, which is also the reason for the high mortality associated with ischemic stroke (3). It is of great importance to understand the mechanisms of neuron injury under ischemic stroke conditions to provide a more precise interference to manage this condition.

Different mechanisms have been reported to be involved in the pathological process of ischemic stroke. Neuron apoptosis (4), necrosis (5) and even ferroptosis (6) have important roles. Of note, disturbed energy supply is reported to be critical to hypoxia-induced neuron death (7). This is understandable, since neurons are highly energy-dependent cells and provides hints that maintaining energy supply may be useful in treating ischemic stroke. In fact, Li *et al* (8) reported that maintaining mitochondrial function prevented nicotine-induced exacerbation of ischemic brain damage, which indicated that mitochondrial function is involved in the pathological processes of ischemic stroke.

As the power factory of the cell, mitochondrial normal function is of importance for neuron survival (9). Mitochondrial dysfunction is involved in several ischemic diseases, including ischemic stroke (10), myocardial infarction (11) and kidney infarction (12). The oxidative phosphorylation processes inside the matrix of mitochondria provides the majority of energy to the cell. However, mitochondria are also vulnerable to stresses and this may result in mitochondrial dysfunction. The present study provided evidence that mitochondrial dysfunction is critically involved in ischemic stroke-induced neuron injury via mitochondrial calcium overload. 75 kDa glucose-regulated protein (GRP75) is a member of the heat shock protein (HSP) family and mediates endoplasmic reticulum (ER)-mitochondrial calcium transfer, maintaining mitochondrial calcium homeostasis (13). However, the role of GRP75 in hypoxia-induced neuron damage has so far remained elusive. The present study examined whether inhibiting GRP75 may ameliorate excessive mitochondrial calcium overload and whether it may be a promising target in preventing hypoxia-induced neuron injury.

## Materials and methods

### Agents

MKT077 (cat. no. HY-15096) was purchased from MedChemExpress. Penicillin-streptomycin mixed solution (cat. no. G4003), pancreatic enzyme (cat. no. G4001), primary antibody diluent (cat. no. G2025), phenylmethylsulphonyl fluoride (PMSF; cat. no. G2008), protein phosphatase inhibitors (cat. no. G2007), DMSO, minimal essential medium (MEM; cat. no. G4553) and 5X loading buffer (cat. no. G2013) were purchased from Wuhan Servicebio Technology Co., Ltd. High-glucose DMEM (cat. no. 10569010), glucose-free DMEM (cat. no. 10966025), Opti-MEM (cat. no. 31985062) and fetal bovine serum (FBS; cat. no. 10091148) were obtained from Gibco (Thermo Fisher Scientific, Inc.). The enhanced chemiluminescence (ECL) kit (cat. no. P0018FS) and bicinchoninic acid (BCA) assay kit (cat. no. P0010S) were purchased from Beyotime Institute of Biotechnology. Primary antibodies against β-actin (cat. no. AC026), GRP75 (cat. no. A112560), mitochondrial fission factor (MFF; cat. no. A8700), mitofusin (MFN)1 (cat. no. A9880), MFN2 (cat. no. A19678), dynamin-1-like protein (DRP1; cat. no. A17069), phosphorylated (p)-DRP1 (cat. no. AP0812) and hypoxia-inducible factor 1-alpha (HIF1α; cat. no. A11945) were purchased from ABclonal Biotech Co., Ltd. Horseradish peroxidase (HRP)-conjugated goat anti-rabbit IgG (cat. no. AS014) and HRP-conjugated goat anti-mouse (cat. no. AS003) were purchased from ABclonal Biotech Co., Ltd. Lipofectamine^®^ 3000 transfection reagent (cat. no. L3000015) and MitoTracker^®^ Deep Red FM kits (cat. no. M7531) were from Thermo Fisher Scientific, Inc.

### Animals

Male C57BL/6 mice (age, 10 weeks; weight, 25–30 g) were purchased from the Animal Experiment Center of Tongji Medical College, Huazhong University of Science and Technology (Wuhan, China). The mice were housed in a specific pathogen-free room with free access to rodent chow and water. The experimental protocol was reviewed and approved by the Animal Research and Care Committee of Tongji Medical College, Huazhong University of Science and Technology [Wuhan, China; no. SYXK(E)2016-0057].

### Mouse middle cerebral artery obstruction (MCAO) model

A mouse model of MCAO was generated as reported previously with minor modifications (14,15). In brief, 14 mice were randomized into the control and MCAO groups (n=7 per group). Individual mice were intraperitoneally injected with 10% chloral hydrate (350 mg/kg) and no peritonitis was present. When the mice were anesthetized, an incision was made in the neck of the mice. After careful exposure of the right external carotid artery (ECA), common carotid artery (CCA) and internal carotid artery (ICA), the CCA was clamped and the ECA was tied, followed by insertion of a 6–0 monofilament nylon suture with a rounded tip from the CCA to the ICA to occlude the left MCA at its origin (~10 mm) and the skin was sutured. The mice were kept on a heating pad to maintain their temperature at 37°C. After recovery from anesthesia, the mice were returned into their cages and they were euthanized at 8 h post-ischemia. The mice were decapitated without anesthesia and four brains were used for 2,3,5-triphenyltetrazolium chloride (TTC) staining and three were analyzed using transmission electron microscopy (TEM).

### TTC staining

The mice were euthanized by decapitation at 8 h of ischemia and their brains were rapidly removed and frozen. The frozen brains were cut into 2-mm sections. The brain slices were stained with 2% TTC solution with incubation at 37°C for 15 min. Subsequently, the brain slices were fixed with 10% paraformaldehyde for 10 min. The percentage of infarct volume of the total brain volume in individual mice was measured using Image J software (v1.51j8) [National Institutes of Health (NIH)] in a blinded manner.

### TEM

The mouse brain tissues were immersed in 2.5% glutaraldehyde (4°C) for 24 h and fixed with 1% osmotic acid-1.5% potassium ferrocyanide for 1.5 h at room temperature. An alcohol gradient was used for dehydration: 75% ethanol for 4 h, 85% ethanol for 2 h, 90% ethanol for 1 h, 100% ethanol for 1 h and another 100% ethanol for 1 h. Next, samples were immersed in 100% xylene for 30 min and 100% xylene for 1 h. Finally, samples were paraffin-embedded for 90 min. Ultrathin tissue sections were stained with 3% uranyl acetate and lead citrate (50 nm) and the mitochondrial morphology was observed by TEM. The length/diameter ratio of the mitochondria was calculated and those with a ratio of >2 were considered as long mitochondria (long-mito) (16).

### Cell culture

HT22 mouse hippocampus neuronal cells (Procell Life Science & Technology Co., Ltd.) and N2a neuroblastoma cells (Wuhan Servicebio Technology Co., Ltd) were cultured in 10% FBS high-glucose DMEM and MEM, respectively, with 1% penicillin and streptomycin (complete medium) in a 5% CO_2_ incubator at 37°C.

### Primary neuron extraction

Primary mouse neuron extraction was performed to investigate mitochondrial function. In brief, one pregnant (E15-17) C57 mouse which was purchased from the Animal Experiment Center of Tongji Medical College, Huazhong University of Science and Technology, was anesthetized using 10% chloral hydrate (350 mg/kg) and the abdominal cavity was quickly opened and fetal mice (six mice) were removed. The pregnant and fetal mice were subsequently decapitated and the cortical regions were isolated and digested with 0.25% trypsin (cat. no. G4001; Wuhan Servicebio Technology Co., Ltd.), followed by termination of digestion with medium (5% FBS neurobasal medium; cat. no. H430717; Shanghai Basalmedia Technologies Co., Ltd.). After centrifugation, the cells were rapidly resuspended in medium. The prepared single cells were cultured in petri dishes coated with polylysine (Gibco; Thermo Fisher Scientific, Inc.). After 12 h of culture, the cells were placed in 1% B27 (FBS-free) medium for subsequent experiments. The experimental protocol was reviewed and approved by the Animal Research and Care Committee of Tongji Medical College, Huazhong University of Science and Technology [approval no. SYXK(E)2017-0012].

### Transfections

The cells were transfected when they had grown to ~70% confluency. For this, 2 µl transfection reagent, 2 µg of probe plasmid or 5 pM of specific small inhibitory (si)RNA were added to 100 µl of Opti-MEM and incubated for 5 min. Opti-MEM with transfection reagents was then added to Opti-MEM with plasmid or siRNA. After resting for 15 min, the mixed suspension was added to the cell culture medium. In addition, a negative control group remained untransfected. The medium was replaced 6 h after transfection, the cells were cultured for another 24 h and then used in the following experiments. The sequences of negative control siRNA (si-NC) and GRP75-specific siGRP75 were as follows: si-NC forward, 5′-UUCUCCGAACGUGUCACGUTT-3′ and reverse, 5′-ACGUGACACGUUCGGAGAATT-3′; siGRP75 forward, 5-GCGUCUUUACCAAACUUAUTT-3′ and reverse, 5′-AUAAGUUUGGUAAAGACGCTT-3′.

### Mitotracker staining

For mitotracker staining, MitoTracker^®^ Deep Red FM kits were used. In brief, cells were stained in the dark using mitotracker (500 nM) for 15 min and then observed with a fluorescence confocal microscope. ImageJ software v.1.51j8 (NIH) was used for the quantification of fluorescence.

### Cellular oxygen and glucose deprivation (OGD) model

The cells were cultured in 10% FBS glucose-free DMEM at 37°C in an incubator with 91% N_2,_ 5% CO_2_ and 4% O_2_ as the OGD conditions for varying time periods. Control cells were cultured under normal cell culture conditions in 95% air and 5% CO_2_; N2a cells were cultured in MEM with 10% FBS and 1% penicillin-streptomycin, while HT22 cells were cultured in DMEM with 10% FBS and 1% penicillin-streptomycin. MKT077 was added to the medium at a final concentration of 5 µM with incubation for 8 h in a 37°C cell incubator.

### Western blot analysis

The different groups of cells were lysed in RIPA buffer containing 1 mM PMSF and 1 mM phosphatase inhibitor cocktail and centrifuged at 14,000 × g for 12 min at 4°C. After determining the protein concentration of each lysate sample using the BCA kit, the lysates (30 µg/lane) were separated by SDS-PAGE on 10–12% gels and transferred onto PVDF membranes (cat. no. WGPVDF45; Wuhan Servicebio Technology Co., Ltd.). The membranes were incubated with 5% dry skimmed milk in 0.1% Tween-20 in Tris-buffered saline for 1 h at room temperature and probed at 4°C overnight with primary antibodies (1:1,000 dilution for all). Subsequently, the membranes were incubated with HRP-conjugated goat anti-rabbit IgG and (1:10,000 dilution) and HRP-goat anti-mouse IgG and visualized with the ECL kit using a Bio-Rad exposure system (Bio-Rad Gel Doc XR; Bio-Rad Laboratories, Inc.) and the Image Lab 5.1 software (Bio-Rad Laboratories, Inc.). Quantitative results were obtained by densitometric scanning using ImageJ v.1.51j8 software (NIH).

### Fluorescent analysis of mitochondrial Ca^2+^

The levels of mitochondrial Ca^2+^ in N2a cells were determined after transfection with mitochondrial Ca^2+^ probe plasmids which had been used previously (17). In each confocal dish, 5×10^4^ N2a cells were cultured for 24 h. The calcium plasmid was transfected into N2a cells for 24 h using the cell transfection method described above. After washing the cells with pre-warmed PBS solution, they were examined under a laser confocal microscope (A1R; Nikon Corporation) using Nikon Imaging Software Elements Viewer 4.20 (Nikon Corporation). The mitochondrial Ca^2+^ levels were determined by measuring average intensity levels using ImageJ software 1.51j8 (NIH).

### Measurement of ATP

The levels of intracellular ATP in individual groups of cells were measured using a specific kit (cat. no. S0026B; Beyotime Institute of Biotechnology) following the manufacturer's protocol. In brief, the same numbers of cells in each group were lysed on ice and centrifuged. The supernatants were collected for the measurement of ATP levels using a luminometer (Promega Corporation) and the levels of ATP were calculated using the standard curve.

### Measurement of the mitochondrial membrane potential (MMP)

The MMP of each group of cells was measured using the specific kit (cat. no. C2006; Beyotime Institute of Biotechnology) following the manufacturer's protocol. In brief, after treatment, the different groups of cells were stained with JC-1 solution at 37°C for 20 min. After being washed, the cells were imaged under a Nikon confocal microscope (A1R; Nikon Corporation) and the red and green fluorescent intensity of 100 cells from 6 randomly selected fields were analyzed using ImageJ software v.1.51j8 (NIH).

### Measurement of reactive oxygen species (ROS) levels

The levels of intracellular ROS were measured using a specific kit (cat. no. S0033; Beyotime Institute of Biotechnology) following the manufacturer's protocol. In brief, the dichloro-dihydro-fluorescein diacetate (DCFH-DA) probe was diluted to 10 mM with FBS-free DMEM. The different groups of cells were stained with 10 mM DCFH-DA in FBS-free DMEM. The fluorescent signals in each group of cells were imaged under a Nikon confocal microscope (A1R; Nikon Corporation) and the green, fluorescent intensity of ~100 cells from 10 randomly selected fields were measured using ImageJ software v.1.51j8 (NIH).

### Statistical analysis

Data were analyzed by GraphPad Prism 5 software (GraphPad Software, Inc.) and are expressed as the mean ± standard error of the mean. Statistical significance was assessed by one-way ANOVA followed by Tukey's post-hoc test. P<0.05 was considered to indicate a statistically significant difference.

## Results

### Mitochondrial fragmentation and dysfunction induced by ischemia in vivo

**Figure 1.**
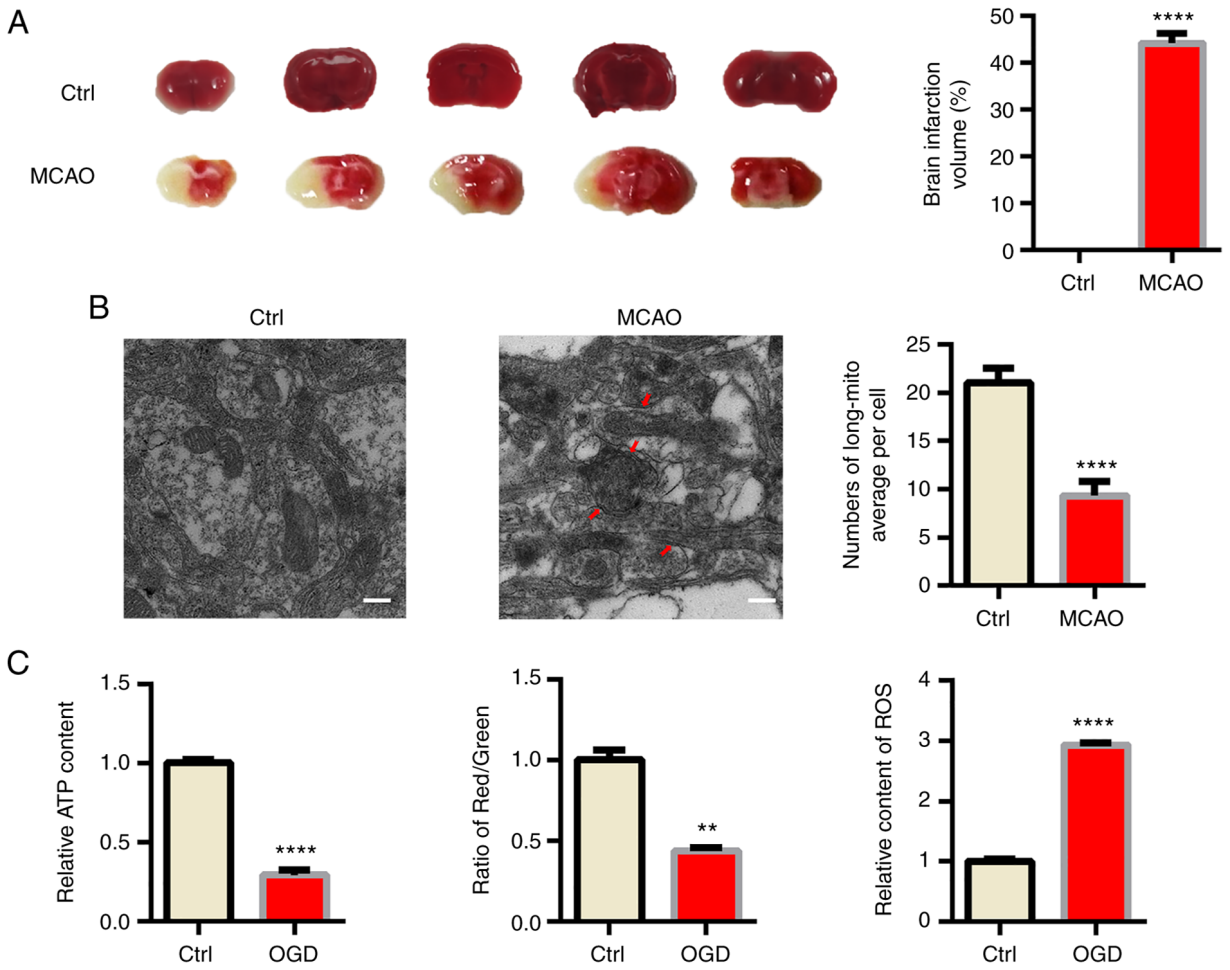
Mitochondria fragmentation and dysfunction induced by ischemia *in vivo* and *in vitro*. (A) Infarct volumes of mouse brains from different groups of mice. (B) Electron microscopy analysis of damaged mitochondrial (arrows) in mouse brains. The number of long mitochondria per cell was determined (scale bars, 2 µm). (C) Primary neurons were cultured under different conditions *in vitro* and the relative ATP content (left panel), mitochondrial membrane potential (relative to red/green fluorescence, middle panel) and relative content of ROS (right panel) were determined. Representative images are provided and quantitative data are expressed as the mean ± standard error of the mean of each group from three separate experiments. **P<0.01, ****P<0.0001 vs. Ctrl. Ctrl, control; MCAO, middle cerebral artery occlusion; OGD, oxygen-glucose deprivation; long-mito, long mitochondria with a length/diameter ratio of >2; ROS, reactive oxygen species.

First, male C57BL/6 mice were subjected to MCAO as a classical *in vivo* model of ischemic stroke (18). TTC staining indicated obvious infraction in the brain of MCAO model mice (Fig. 1A). Acute hypoxia significantly influenced mitochondrial function; hence, TEM was used to confirm the morphology changes of mitochondria in ischemic mouse brains. As indicated in Fig. 1B, swollen and fragmented mitochondria were apparent in the MCAO group and the cristae of mitochondria were broken and fractured. However, the morphology of mitochondria was normal in the sham mice. Since mitochondrial function is tightly associated with the morphology of mitochondria (19), several indexes reflecting mitochondrial function, including ATP content, ROS production and the MMP were also determined and it was indicated that mitochondrial function was significantly impaired by OGD (Figs. 1C, S1 and S2).

### Mitochondrial fragmentation and dysfunction induced by OGD in vitro

**Figure 2.**
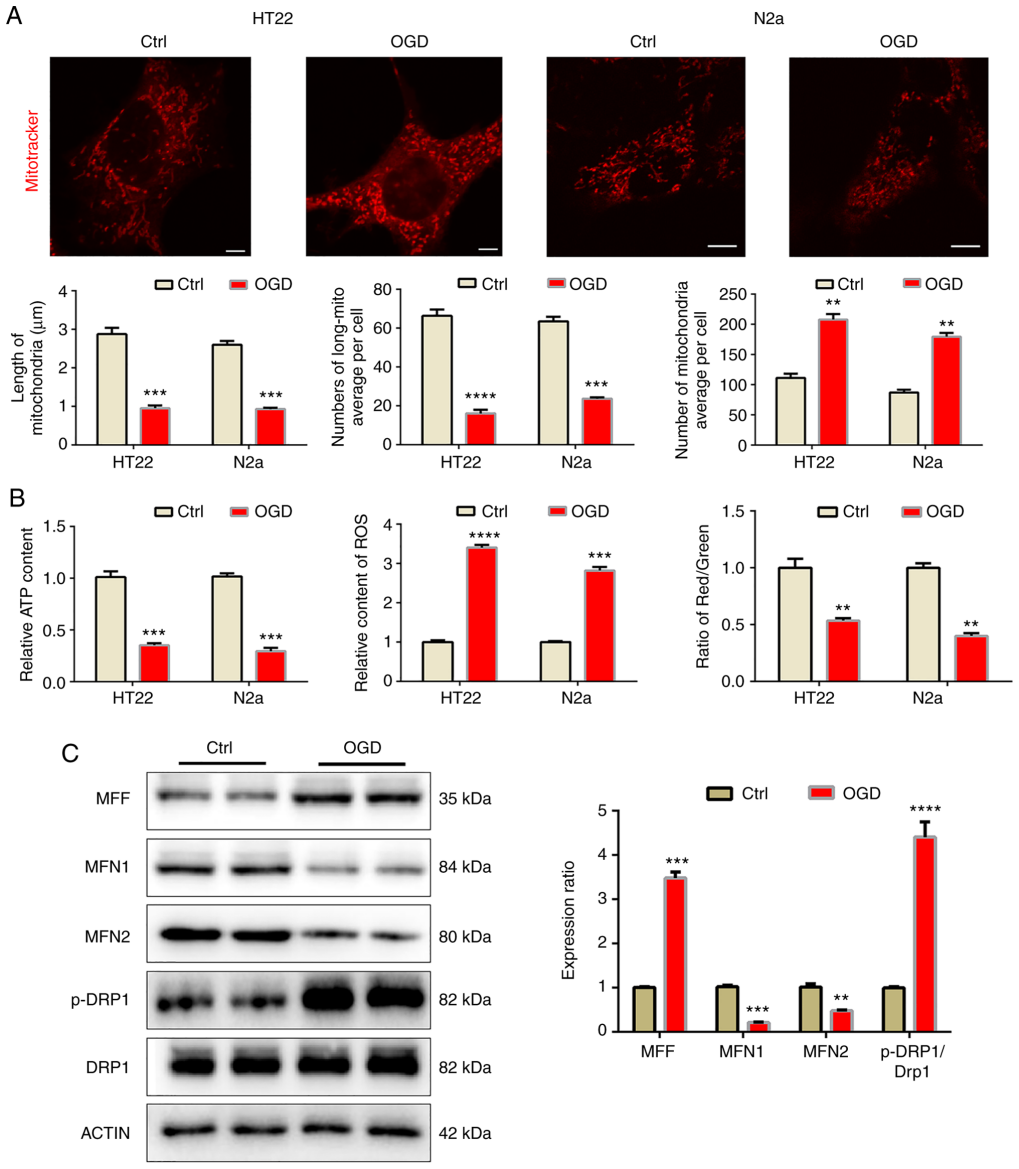
Mitochondrial fragmentation and dysfunction induced by OGD *in vitro*. (A) Fluorescence imaging analysis of mitochondrial morphology of HT22 and N2a cells (scale bars, 5 µm). (B) Western blot analysis revealed mitochondrial dynamic protein expression in the N2a cells. (C) OGD impaired mitochondrial function in HT22 and N2a cells. Representative images are provided and quantitative results are expressed as the mean ± standard error of the mean of each group from three separate experiments. **P<0.01, ***P<0.001, ****P<0.0001 vs. Ctrl. Ctrl, control; OGD, oxygen-glucose deprivation; MFN, mitofision protein; MFF, mitochondria fission factor; DRP1, dynamin-1 protein; p, phosphorylated.

In order to further confirm the above results in an *in vitro* model, OGD was implemented to resemble the effects of MCAO in the HT22 and N2a cell lines. After 8 h of OGD, cellular mitochondria were observed by mitotracker staining (Fig. 2A). The mitochondria became fragmented after OGD, as the length of mitochondria decreased while the number of mitochondria per cell increased after OGD, which led to a decreased mitochondrial ratio. Similarly, OGD also impeded mitochondrial function (Fig. 2B) and decreased the MMP and ATP levels but increased ROS levels. In N2a cells, western blot analysis was used to detect mitochondrial dynamic-related proteins. The results indicated that under OGD conditions, mitochondrial fusion proteins MFN1 and MFN2 were downregulated, while mitochondrial fission proteins MFF and p-DRP1 increased significantly, indicating that the mitochondria became fragmented (Fig. 2C).

### Mitochondrial calcium overload induced by OGD

**Figure 3.**
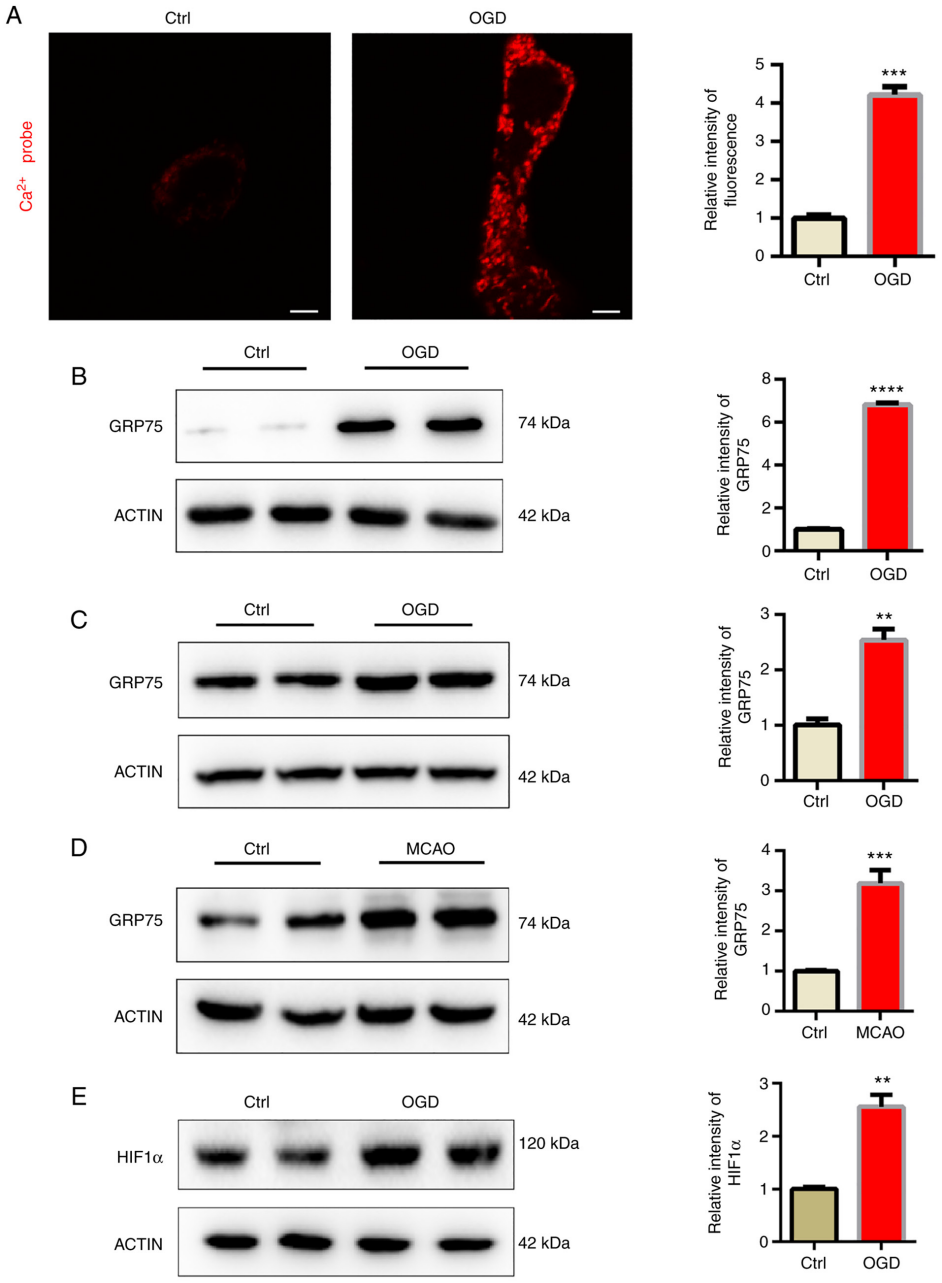
Mitochondrial calcium overload induced by OGD. (A) OGD-stimulated calcium overload in the mitochondria of N2a cells (scale bars, 5 µm). (B and C) Western blot analysis revealed that OGD upregulated GRP75 expression in (B) HT22 and (C) N2a cells. (D) Western blot analysis revealed that OGD upregulated GRP75 expression in the MCAO tissue. (E) Western blot analysis was used to determine HIF1α expression in N2a cells. Representative images are provided and quantitative results are expressed as the mean ± standard error of the mean of each group from three separate experiments. **P<0.01, ***P<0.001, ****P<0.0001 vs. Ctrl. GRP75, 75 kDa glucose-regulated protein; Ctrl, control; OGD, oxygen-glucose deprivation; MCAO, middle cerebral artery occlusion; HIF, hypoxia-inducible factor.

GRP75 acts as a bridge, linking the ER-anchored inositol 1,4,5-trisphosphate receptor type 1 (IP3R) and mitochondria-located voltage-dependent anion-selective channel protein (VDAC) to form a protein complex and induce calcium transfer between ER and mitochondria (13). In the present study, a mitochondrial tagged calcium sensor vector was used to monitor calcium levels in mitochondria and it was indicated that mitochondrial calcium levels were significantly elevated after OGD (Fig. 3A). It was then examined whether mitochondrial overload was related to abnormal GRP75 expression. Consistent with the present hypothesis, GRP75 was significantly elevated after OGD (Fig. 3B and C). This indicated that GRP75 may be related to OGD-induced mitochondrial calcium overload. The expression of GRP75 in the infarction area of the MCAO model was also examined, suggesting that GRP75 was also elevated *in vivo* (Fig. 3D). HIF1α is one of the most important modulators of hypoxia and the expression of HIF1α was thus determined; the results indicated that HIF1α was upregulated (Fig. 3E). Therefore, it may be hypothesized that the increase of HIF1α induced the upregulation of GRP75.

### Inhibition of GRP75 by pharmacological agent preserves mitochondrial morphology and function under OGD

**Figure 4.**
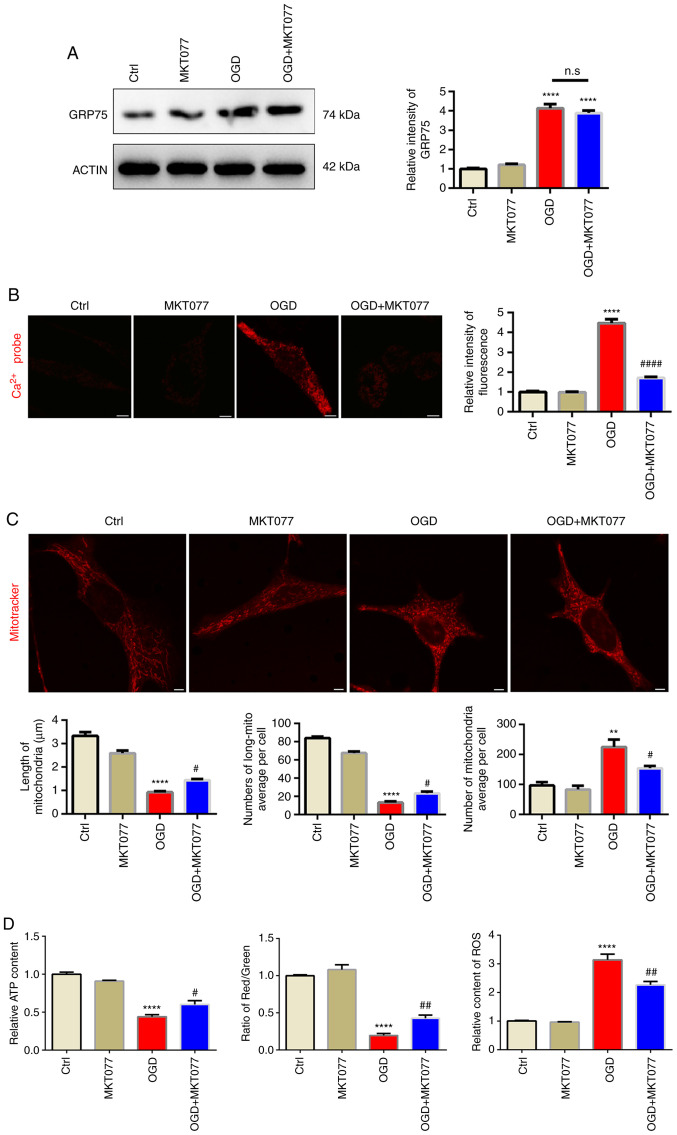
Inhibition of GRP75 by pharmacological agent preserves mitochondria morphology and function under OGD. (A) Western blot analysis revealed that MKT077 regulated GRP75 expression in HT22 cells. (B) Treatment with MKT077 to inhibit GRP75 mitigated the OGD-stimulated calcium overload in the mitochondria of Na2 cells. (C) Fluorescence imaging analysis of mitochondrial morphology of HT22 cells (scale bars, 5 µm). (D) Mitochondrial function in N2a cells from the different groups. Representative images are provided and quantitative data are expressed as the mean ± standard error of the mean of each group from three separate experiments. **P<0.01, ****P<0.0001 vs. Ctrl; ^#^P<0.05, ^##^P<0.01, ^####^P<0.0001 vs. OGD group. n.s., no significance; GRP75, 75 kDa glucose-regulated protein; Ctrl, control; OGD, oxygen-glucose deprivation.

After confirming abnormal elevation of GRP75 after OGD, it was investigated whether interfering with GRP75 is an effective strategy to protect mitochondrial function after OGD. MKT077, a GRP75-specific inhibitor (20), which is able to bind to GRP75 and abrogate its activity (21), was applied and it was observed that, although MKT077 did not downregulate GRP75 expression after OGD (Fig. 4A), mitochondrial calcium overload was obviously inhibited after MKT077 treatment (Fig. 4B), which indicated that inhibiting GRP75 function prevented mitochondrial calcium overload. As expected, mitochondrial morphology was well-preserved by MKT077 even after OGD (Fig. 4C). This further supported the notion that mitochondrial calcium overload was able to trigger mitochondrial fragmentation and induce mitochondrial dysfunction (Figs. 4D, S1 and S2).

### Knockdown of GRP75 preserves mitochondrial morphology and function under OGD

**Figure 5.**
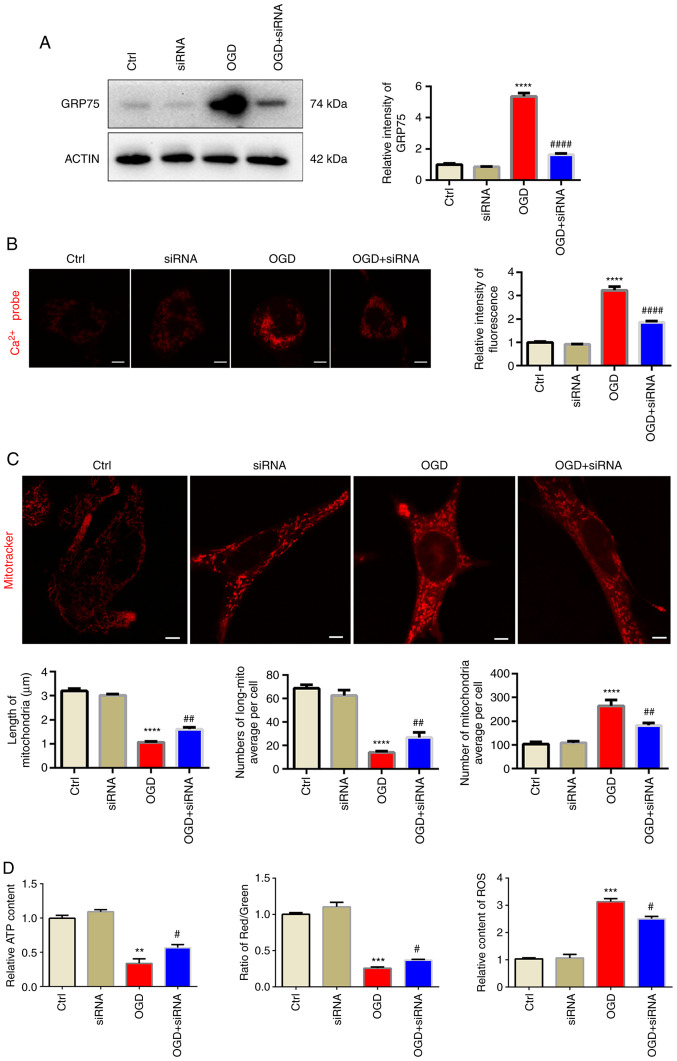
Knockdown of GRP75 preserves mitochondrial morphology and function under OGD. (A) Western blot analysis confirmed siRNA-mediated knockdown of GRP75 expression in N2a cells. (B) Treatment with siRNA targeting GRP75 to inhibit GRP75 mitigated the OGD-stimulated calcium overload in the mitochondria of N2a cells. (C) Fluorescent imaging analysis of mitochondrial morphology of N2a cells (scale bars, 5 µm). The average length of mitochondria, number of long mitochondria per cell and average number of mitochondria per cell were determined. (D) Mitochondrial function in N2a cells from the different groups; the relative ATP content (left panel), mitochondrial membrane potential (relative to red/green fluorescence, middle panel) and relative content of ROS (right panel) were determined. Representative images are provided and quantitative data are expressed as the mean ± standard error of the mean of each group from three separate experiments. **P<0.01, ***P<0.001, ****P<0.0001 vs. Ctrl; ^#^P<0.05, ^##^P<0.01, ^####^P<0.0001 vs. OGD group. Ctrl, control; OGD, oxygen-glucose deprivation; GRP75, 75 kDa glucose-regulated protein; siRNA, small interfering RNA; long-mito, long mitochondria with a length/diameter ratio of >2; ROS, reactive oxygen species.

In order to exclude any possible off-target effects of the small molecule antagonist, siRNA targeting at GRP75 was used to further confirm its effect on mitochondrial overload. siGRP75 efficiently reduced GRP75 expression levels after OGD, while si-NC did not influence the expression of GRP75 (Figs. 5A and S3). Similar to the effect of MKT077, siGRP75 was also able to reduce mitochondrial calcium overload (Fig. 5B) and preserve mitochondrial morphology and function after OGD (Figs. 5C and D, S1 and S2), which further confirmed that inhibiting GRP75 preserved mitochondrial morphology and function.

## Discussion

As mitochondria are the major energy-producing organelles, their normal function is vital to cell survival, particularly to cells with high energy demand, such as neurons or cardiomyocytes (22). In the present study, it was indicated that mitochondrial dysfunction took place both *in vivo* and *in vitro* under hypoxia stress. GRP75 was significantly upregulated by MCAO *in vivo* or OGD *in vitro*, and by inhibiting GRP75 via antagonist or siRNA, it was further confirmed that GRP75 was key to mitochondrial overload triggered by hypoxia. The present study supports the idea that GRP75 may be a promising target in treating ischemic stroke in future research.

Ischemic stroke affects the quality of life of patients. Neurons are vulnerable to lack of oxygen (23), which may cause dysfunction of mitochondria, the power factory of the cell, leading to various neuronal disturbances. Qadri *et al* (24) reported that mitochondrial dysfunction was one of the important causes of Parkinson's disease. Zhao *et al* (25) indicated that after MCAO treatment, the expression of mitochondrial fission protein increased while the expression of mitochondrial fusion protein decreased, indicating that mitochondrial fragmentation occurred in the MCAO model, resulting in mitochondrial dysfunction and affecting the activity of neurons. Therefore, it is essential to maintain normal function of mitochondria to protect neurons.

GRP75 belongs to the mammalian HSP70 family, which is highly conserved and has various cellular functions, including cell survival (26), the cell cycle and apoptosis (27). It can help maintain intracellular homeostasis under various stresses, including hypoxia (28). However, GRP75 has another important cellular function as a ‘molecular bridge’ between IP3R and VDAC to form a calcium channel between the ER and mitochondrion. Although calcium is required by normal mitochondrial oxidative phosphorylation, excessive calcium disturbs this process and trigger more ROS production (29). Calcium overload is thought to be an important pathological process during several ischemic diseases such as stroke and myocardial infarction, under which circumstances excessive calcium floods into the mitochondrion through different calcium channels, leading to mitochondrial dysfunction (30). In the present study, it was indicated that under the conditions of OGD, the expression of GRP75 in neurons increased significantly and the mitochondria appeared to have calcium overload. This indicates that calcium flooded into the mitochondria. GRP75 is an important component in the OGD model, resulting in mitochondrial calcium overload and mitochondrial damage. Furthermore, evidence was provided that inhibiting GRP75 via MKT077 or direct knockdown of GRP75 alleviated OGD induced mitochondrial calcium overload and preserved cell viability. However, as a member of the HSP family, GRP75 also has various physiological functions, such as iron-sulfur cluster assembly, protein refolding (31) and erythrocyte differentiation (32). Considering such important physiological functions of GRP75, how to titer optimized dosage of GRP75 inhibition under different stress conditions may be a challenge and this is the obvious weakness of inhibiting GRP75 and requires further investigation. In conclusion, GRP75 may be a promising target but requires further investigation.

MKT-077 is a rhodacyanine dye and also an HSP70 inhibitor, which exhibits significant antitumor activity (33). A previous study by our group indicated that MKT077 did not reduce the expression of GRP75 but inhibited the GRP75-mediated ER-mitochondrial calcium exchange (16). Miyata *et al* (34) reported that a derivative of MKT077 had blood-brain-barrier permeability properties and reduced Tau levels in the brain. Thus, further studies by our group will use YM-08 (a derivative of MKT077) for intragastric administration of mice to observe its therapeutic effect. This noteworthy pharmacological evolution of MKT077 provided evidence that GRP75 was able to regulate cellular processes more than maintaining calcium homeostasis. However, concerns about off-target effects of small-molecule antagonists led us to use siRNA to more precisely interfere with GRP75 and the results further confirmed the function of GRP75 in the pathophysiological process of ischemic stroke.

However, there are several limitations of the present study. Mitochondrial function may only be roughly evaluated via the MMP, as well as ROS and ATP production. Furthermore, mitochondrial calcium was only determined in N2a cells, while it was not possible to measure calcium ions in the mitochondria of HT22 cells due to issues with the transfection of the calcium probe plasmid. In addition, the mechanism by which ischemic stress increases GRP75 expression remains elusive. Under hypoxia circumstances, HIF1α is rapidly upregulated and is able to regulate the expression of various genes. It was reported that HIF1α was able to regulate the expression of GRP75 (35); hence, it may be the mechanism of GRP75 upregulation by MCAO or OGD in the present study.

**Figure 6.**
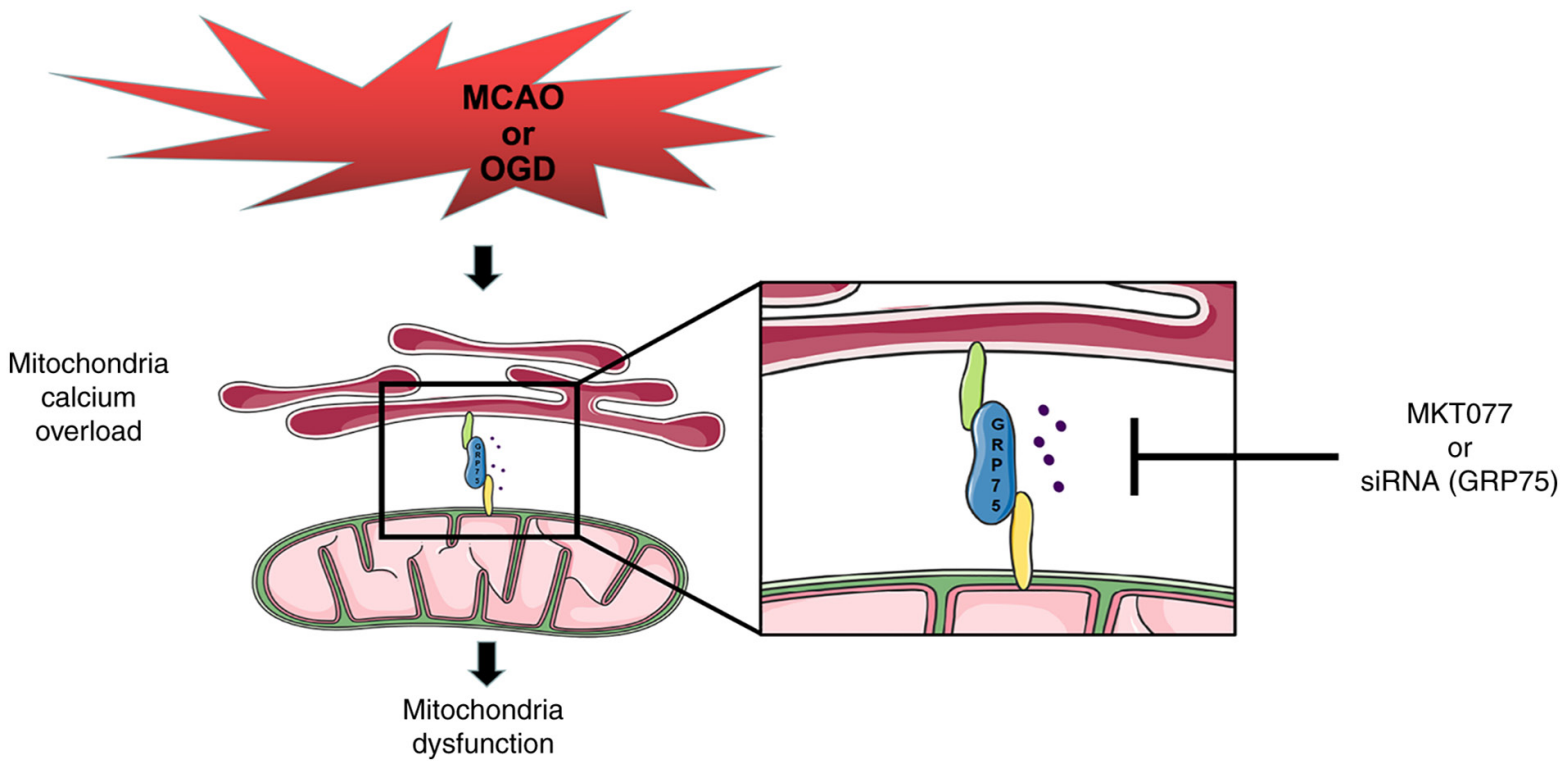
Schematic illustrating that preserving mitochondrial function by inhibiting GRP75 ameliorates neuron injury under ischemic stroke. Ischemia or OGD may also upregulate GRP75 expression to promote mitochondrial calcium overload, thus causing mitochondrial dysfunction and neuron death. Treatment with MKT077 or siRNA may inhibit GRP75 activation or expression and attenuate the ischemia- or OGD-induced mitochondrial calcium overload in neurons. GRP75, 75 kDa glucose-regulated protein; siRNA, small interfering RNA; OGD, oxygen-glucose deprivation; MCAO, middle cerebral artery occlusion.

In conclusion, the present study provided evidence that GRP75 is an important factor in hypoxia-induced neuron injury both *in vivo* and *in vitro*. GRP75 was able to augment mitochondria-associated endoplasmic reticulum membranes formation and lead to mitochondrial calcium overload, ultimately leading to mitochondrial dysfunction and disruption of energy supply to neurons, making neurons vulnerable to hypoxia (Fig. 6). Inhibiting GRP75 may be a promising strategy to ameliorate injury following ischemic stroke in future research.

## Supplementary Material

Supporting Data

## Data Availability

The raw data and materials generated and used during this study are available from the corresponding author upon reasonable request.
