# Supplementary material for: Preserving mitochondrial function by inhibiting GRP75 ameliorates neuron injury under ischemic stroke
Source: Mol Med Rep. 2022 Mar 15;25(5):165. doi: 10.3892/mmr.2022.12681 (PMC8941507; doi:10.3892/mmr.2022.12681)

Figure S1. Continued.

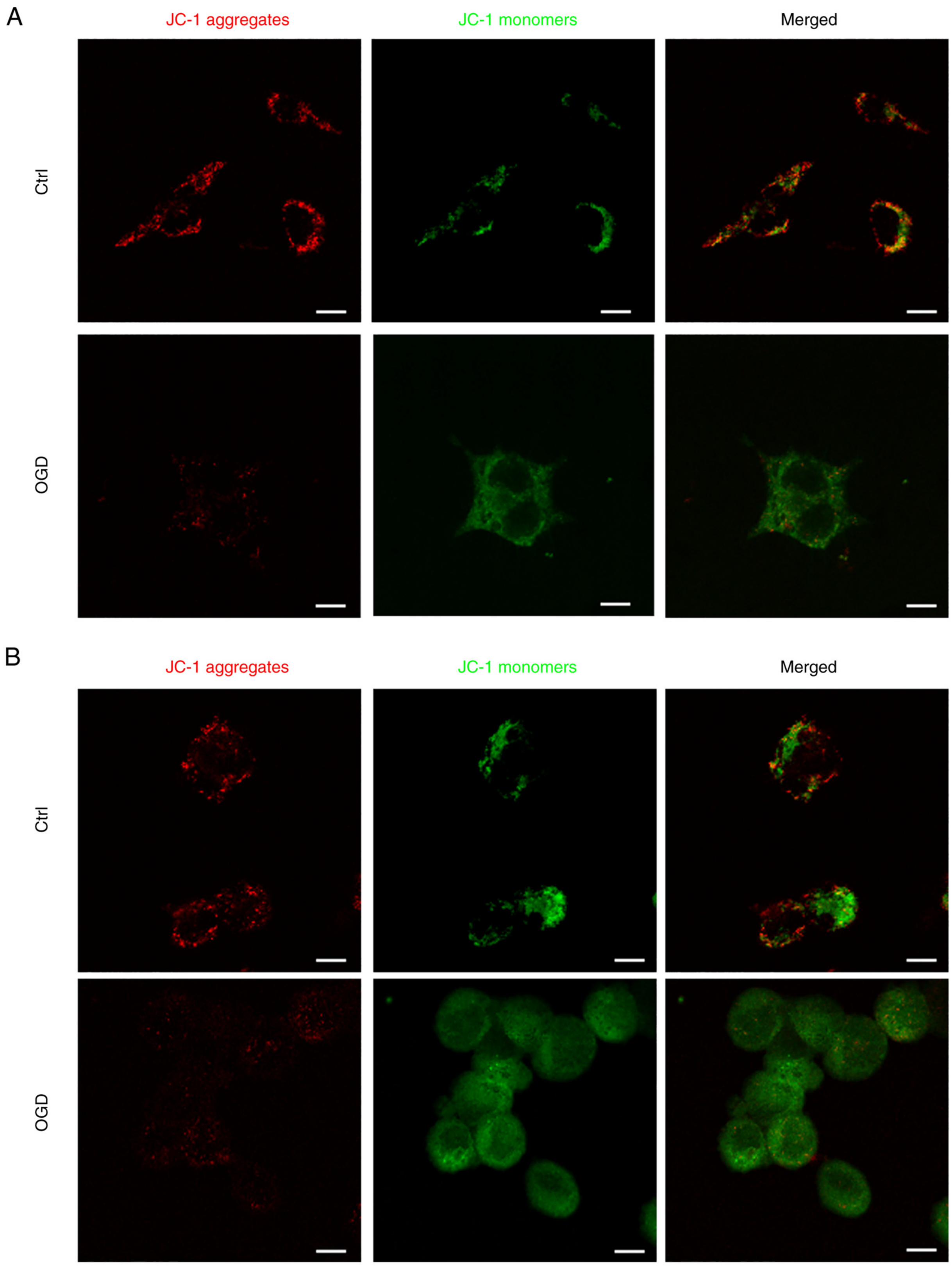

Figure S1. Continued.

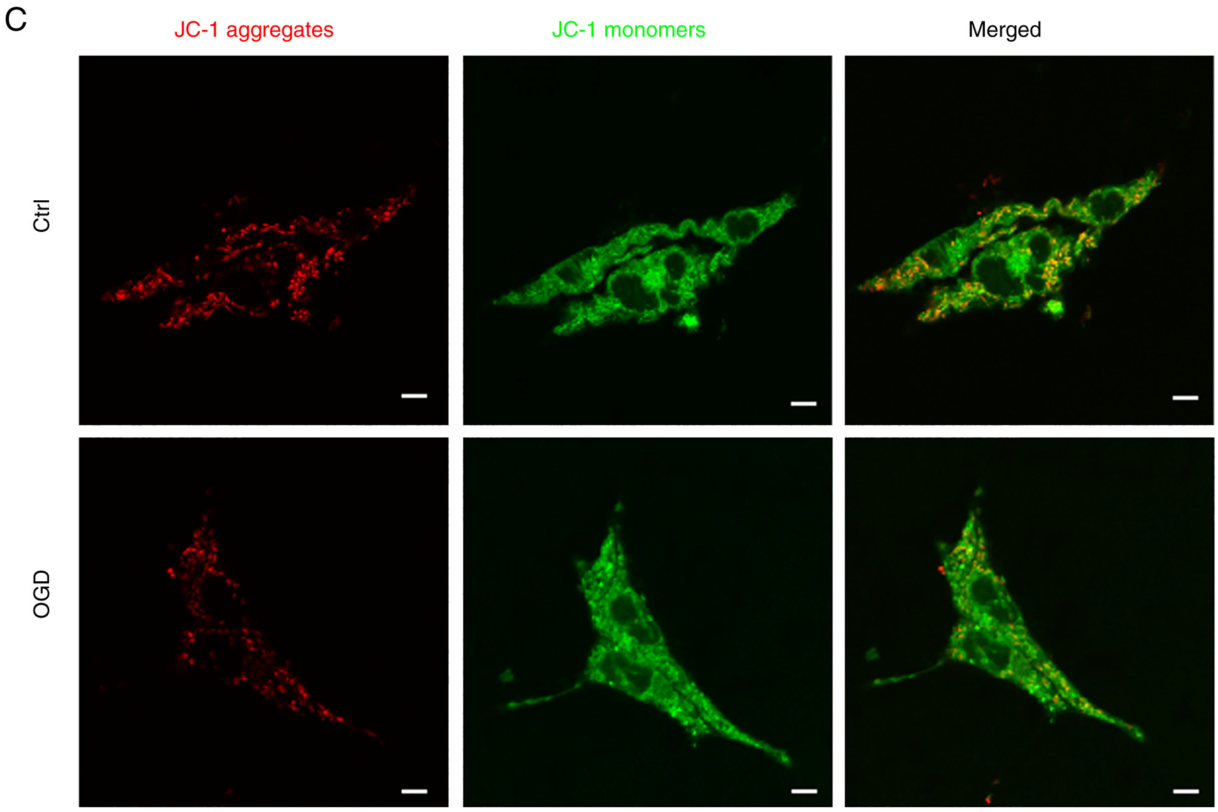

Figure S1. (A) Microscopy images of JC-1 staining of primary neurons as in Fig. 1C; (B) microscopy images of JC-1 staining of N2a as in Fig. 2B; (C) microscopy images of JC-1 staining of HT22 as in Fig. 2B; (D) microscopy images of JC-1 staining of N2a as in Fig. 4D; (E) microscopy images of JC-1 staining of N2a as in Fig. 5D. Representative images are provided (scale bars, 5 μm). Ctrl, control; OGD, oxygen-glucose deprivation.

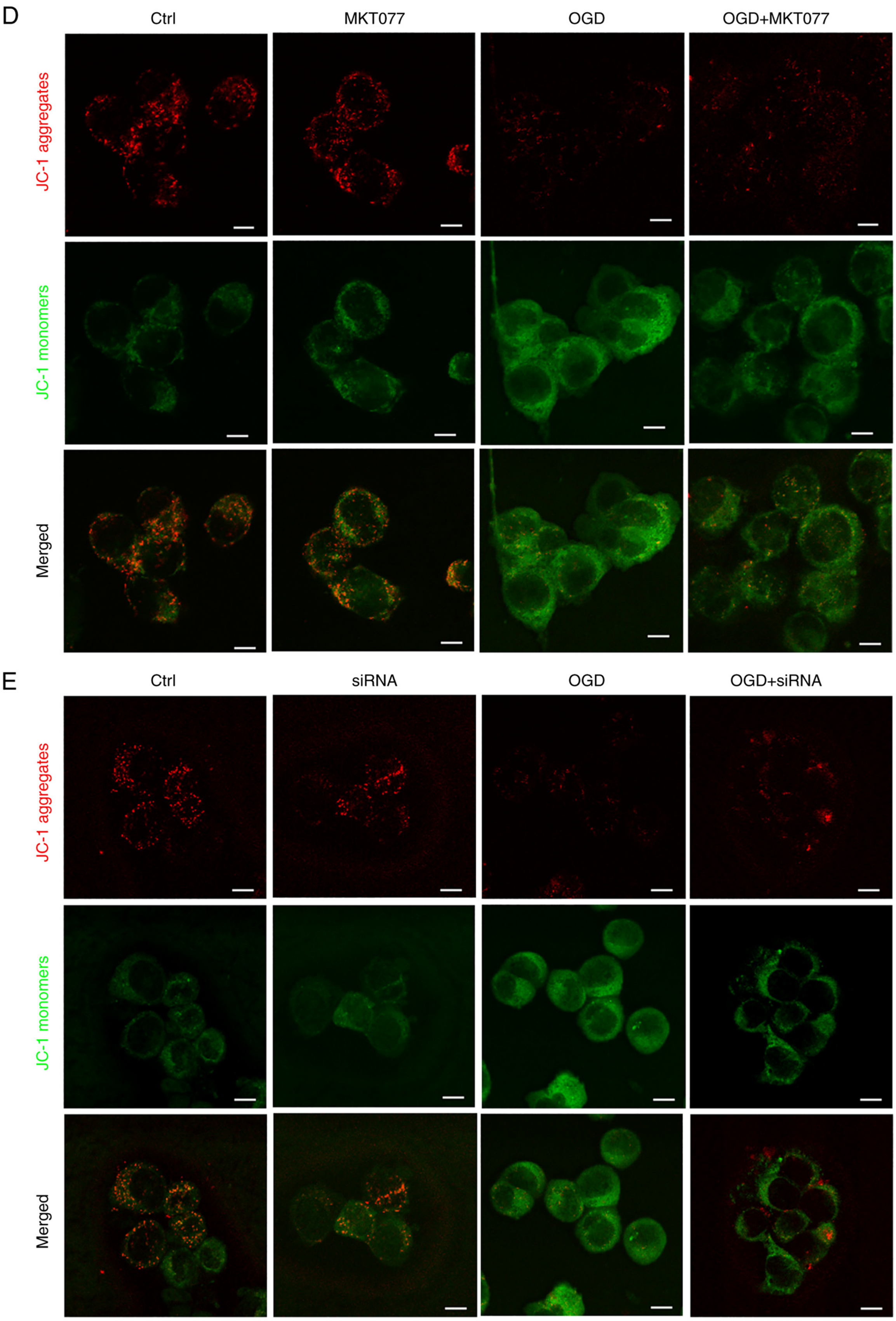

Figure S2. (A) Microscopy images of DCFH-DA staining of primary neurons as in Fig. 1C; (B) microscopy images of DCFH-DA staining of N2a as Fig. 2B; (C) microscopy images of DCFH-DA staining of HT22 as in Fig. 2B; (D) microscopy images of DCFH-DA staining of N2a as in Fig. 4D; (E) microscopy images of DCFH-DA staining of N2a as in Fig. 5D. Representative images are provided (scale bars, 5 μm). Ctrl, control; OGD, oxygen-glucose deprivation; DCFH-DA, dichloro-dihydro-fluorescein diacetate.

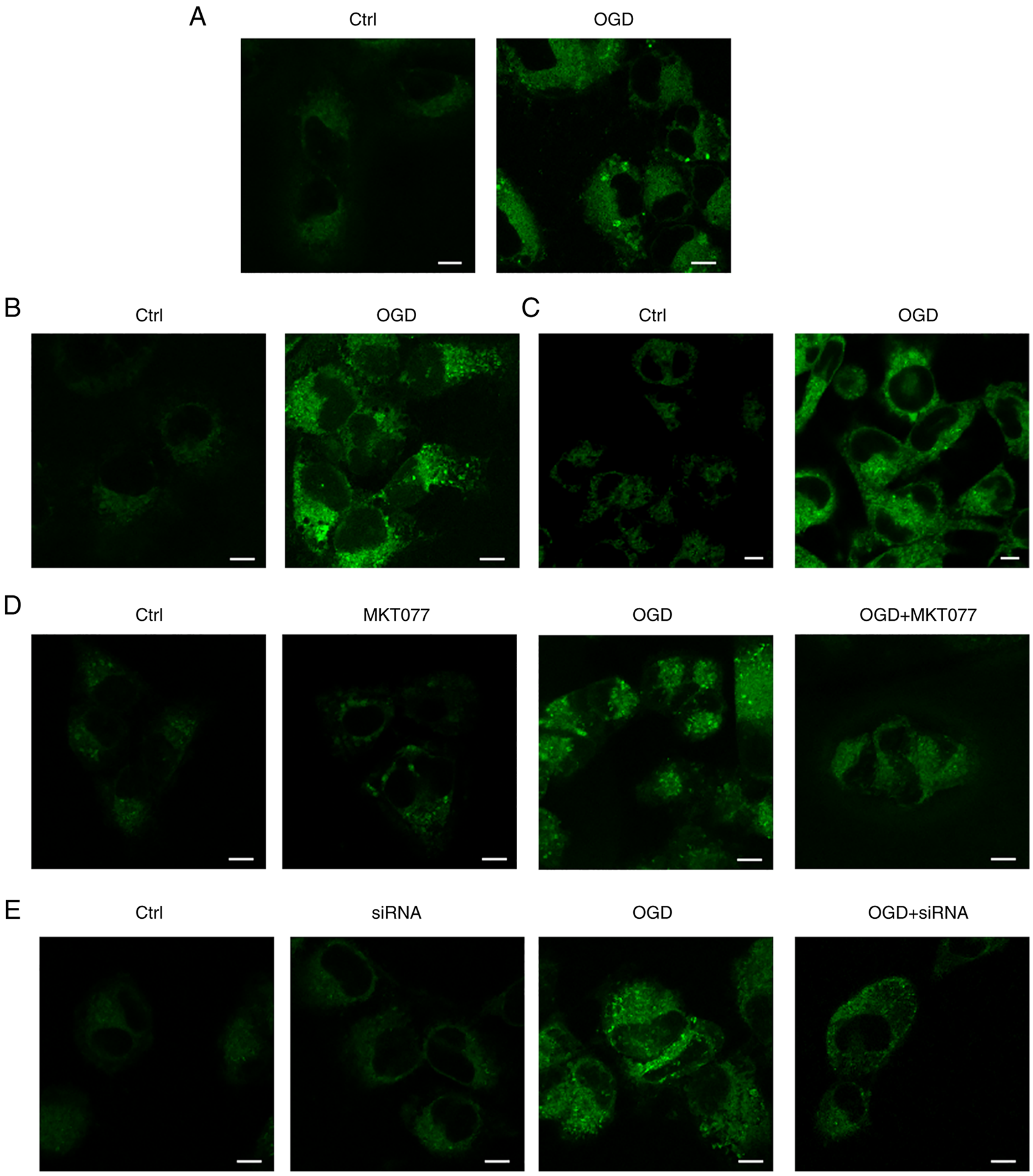

Figure S3. Western blot analysis revealing that si-NC and siGRP75 regulate GRP75 expression in N2a cells. Representative images are provided and quantitative data are expressed as the mean ± standard error of the mean of each group from three separate experiments. ****P<0.0001. n.s., no significance; GRP75, 75 kDa glucose-regulated protein; siRNA, small inhibitory RNA; si-NC, negative control siRNA; siGRP75, siRNA targeting GRP75.

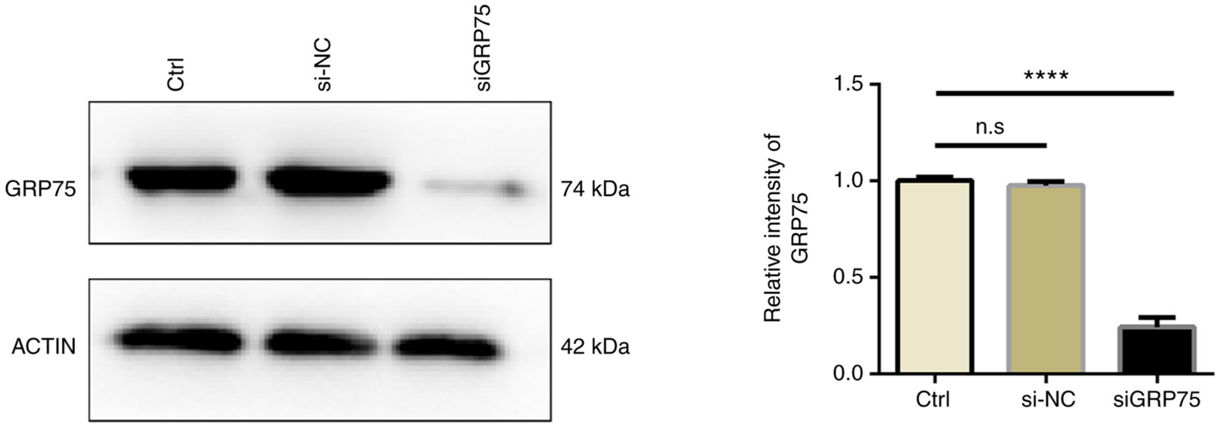

Supplement: Supporting Data [file Supplementary_Data.pdf]
